# Understanding the Effects of Radiotherapy on the Tumour Immune Microenvironment to Identify Potential Prognostic and Predictive Biomarkers of Radiotherapy Response

**DOI:** 10.3390/cancers12102835

**Published:** 2020-09-30

**Authors:** Shuhui Cheng, Eleanor J. Cheadle, Timothy M. Illidge

**Affiliations:** 1 Manchester Academic Health Science Centre, Manchester NIHR Biomedical Research Centre, Division of Cancer Sciences, Faculty of Biology, Medicine and Health, University of Manchester, Manchester M13 9PL, UK; shuhui.cheng@postgrad.manchester.ac.uk (S.C.); Eleanor.J.Cheadle@manchester.ac.uk (E.J.C.); 2 The Christie NHS Foundation Trust, Manchester M20 4BX, UK

**Keywords:** radiotherapy, immunotherapy, tumour microenvironment, biomarker

## Abstract

**Simple Summary:**

Around 50% of all cancer patients receive radiotherapy as part of their treatment. Recently immunotherapy has shown promising results and become established as an effective treatment for some cancers. Combining radiotherapy and immunotherapy is a novel approach to further increase the number of patients responding to immunotherapy. Biological markers of response (biomarkers) are urgently required to hasten the clinical translation and improve outcomes further. Radiotherapy can both stimulate and inhibit the immune system and understanding the immune effects of radiotherapy on the tumour and surrounding cells may lead to the identification of predictive and prognostic biomarkers to help make more individualized treatment decisions, when combining radiotherapy with immunotherapy. This review summarizes the immune effects of radiotherapy and biomarkers of response identified to date; providing new perspectives for future research which may facilitate the development of novel radiotherapy immunotherapy combinations based on tumour immunology and biomarker identification.

**Abstract:**

Radiotherapy (RT) is a highly effective anti-cancer treatment. Immunotherapy using immune checkpoint blockade (ICI) has emerged as a new and robust pillar in cancer therapy; however, the response rate to single agent ICI is low whilst toxicity remains. Radiotherapy has been shown to have local and systemic immunomodulatory effects. Therefore, combining RT and immunotherapy is a rational approach to enhance anti-tumour immune responses. However, the immunomodulatory effects of RT can be both immunostimulatory or immunosuppressive and may be different across different tumour types and patients. Therefore, there is an urgent medical need to establish biomarkers to guide clinical decision making in predicting responses or in patient selection for RT-based combination treatments. In this review, we summarize the immunological effects of RT on the tumour microenvironment and emerging biomarkers to help better understand the implications of these immunological changes, and we provide new insights into the potential for combination therapies with RT and immunotherapy.

## 1. Introduction

Radiotherapy (RT) is a highly effective anti-cancer treatment delivered to between 50% and 60% of all cancer patients as part of either curative treatment or for palliation of their disease [1]. Following the breakthrough of immune checkpoint inhibitors (ICI), immunotherapy has now become established as an important component of cancer therapy. However, the response rate to single agent ICI in most solid tumours is low, at approximately 20% to 30%, and further developments are ongoing to improve response rates and outcomes further. RT has long since been known to be highly effective at inducing DNA damage and in recent years has been shown to have local and systemic immunomodulatory effects. Therefore, combining RT and immunotherapy is a logical approach to enhance anti-tumour immune responses. However, the immunomodulatory effects of RT can be both immunostimulatory and immunosuppressive, which may be different across different tumour types and patients. This potential diversity in the immune response to RT adds complexity to the interpretation of how the immune landscape in the tumour microenvironment (TME) might respond in patients receiving RT. The nature of the immune response to RT and whether this is immunostimulatory or immunosuppressive further highlights the importance of identifying immunological biomarkers to assess treatment responses in patients undergoing RT. In this review, the effects of RT on the tumour microenvironment, candidate immune targets, emerging biomarkers and the related cutting-edge technologies for analysis of immune biomarkers will be outlined. Finally, the implications of these immunological consequences on the potential for combination therapy with RT and immunotherapy will be discussed.

## 2. The Tumour Immune Microenvironment

### 2.1. The Components and Classification of TME

The term “tumour microenvironment” (TME) refers to an “ecological niche” which affects tumour growth and progression and is characterized by complex biological interactions between the tumour and the stroma [2,3]. The TME encompasses cancer cells, stromal cells (such as fibroblasts), a diversity of resident and infiltrating immune cells and soluble messengers [2]. The constituent cell types in the TME vary from T cells, dendritic cells (DCs), tumour-associated macrophages (TAMs), myeloid-derived suppressor cells (MDSCs), mast cells, to natural killer (NK) cells, which secrete a variety of factors (chemokines, cytokines and enzymes) that directly or indirectly participate in immune responses. The composition and the interplay of the components within the TME may have a critical role in the outcome of tumour evolution and treatment response [2].

There are certain cell types in the TME that play an essential role in immune surveillance, such as DCs, cytotoxic T cells and NK cells. Among these, the T cell infiltrates are potentially some of the most important immune effector cells populations which have been investigated extensively. One such investigation is the immunoscore, which refers to the density of two different lymphocyte populations (CD3+, CD8+ or CD45RO+ cells) quantified in the tumour core and the invasive margin, which was first shown to correlate with outcome in colorectal cancer (CRC) [4]. The prognostic power of the immunoscore is also being investigated and validated in other cancer types including melanoma and breast cancer [5].

The TME can however become highly immunosuppressive as a result of the infiltration and interaction of a multitude of immune effector cells which act to suppress immune responses, including MDSCs, regulatory T cells (Tregs) and TAMs. Tregs play a significant role in facilitating immune escape and promoting tumour progression. A low number of Tregs before treatment was found to be correlated with active immune responses and favourable clinical outcome in breast cancer patients following surgery and RT [6]. In terms of TAM, the TME encourages the switch from a tumour-killing M1-TAM to a tumour-promoting M2-TAM type. MDSCs are a heterogeneous population of myeloid cells [7] and have been demonstrated to be associated with a poor prognosis in pancreatic, oesophageal and gastric cancers, which suggests their potential as an important prognostic biomarker [7].

It is increasingly recognized that tumour responses to treatments to some extent depend on the microenvironment. Therefore, it is important to understand how immunostimulatory and suppressive effects are reflected and balanced in the TME, thereby hopefully tailoring the treatment according to the TME landscape.

### 2.2. The Effects of RT on the Tumour Microenvironment

RT has been widely used to eradicate cancer with its direct tumour-killing effects [8]; however, increasing evidence shows that RT can cause tumour rejection by enhancing local anti-tumour immune responses that can lead to a distant systemic immune response called the “abscopal effect”, where the tumour regresses outside the irradiated site [9]. However, it is increasingly understood that RT has both immunostimulatory and immunosuppressive effects. In order to utilize RT to enhance the immunostimulatory effects and overcome intrinsic immunosuppression within the TME, a greater understanding of RT-induced immune effects is required.

#### 2.2.1. RT-Induced Immunostimulation

RT is well documented to initiate tumour rejection by enhancing anti-tumour immune responses, although mostly in murine tumours [10,11,12,13,14,15,16]. The phenomenon “abscopal effect”, which refers to a tumour in a non-irradiated site regressing after RT [17], offers further evidence of the ability of RT to induce immunological responses. RT can mediate these effects in several ways including immunogenic effects on tumour cells and immunostimulatory effects on the immune system.

RT can increase the antigenicity of tumour cells by inducing or upregulating the release of tumour-associated antigen (TAA) through induction of tumour cell death and increasing the expression of MHC class I molecules that are expressed on the surface of tumour cells. RT can also greatly alter the repertoire of MHC class I restricted peptides [18].

**Figure 1:**
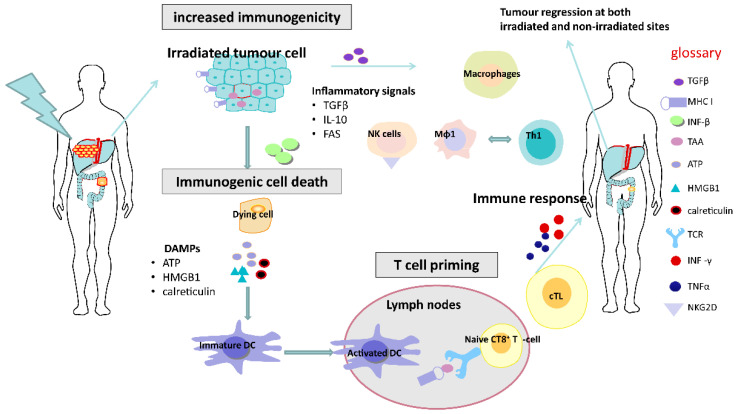
Immunostimulatory effects of radiotherapy. Radiotherapy can enhance the antigenicity of tumour cells by stimulating the induction of immunogenic cell death mediated by damage-associated molecular pattern molecules (DAMPs), which contributes to DC maturation and T cell priming. Activated cytotoxic T cells (cTL) in the blood can cause systemic immune responses, potentially resulting in tumour regression outside the irradiated field. DC, dendritic cell; Mφ1 cells, macrophage type-1 cell; Th1 cell, T helper type 1 cell.

It has been shown that RT is able to induce immunogenic cell death (ICD), whereby tumour cells dying after RT undergo a form of cellular death induced by a cascade of complex reactions which can elicit immune responses [19] (Figure 1).

After irradiation, tumour cells can undergo a form of cell death known as immunogenic cell death and express damage-associated molecule patterns (DAMP) or “danger signals”, which includes exposure of calreticulin, the extracellular release of ATP and high mobility group box 1 (HMGB1) and uric acid. Calreticulin is a protein that can serve as an “eat me” signal, stimulating the engulfment of dying tumour cells and their apoptotic debris by macrophages and immature DCs [19,20]. ATP can be a potent signal for activating myeloid cells including monocytes/macrophages and immature DCs. HMGB1 is a factor that can be released by dead cells and can interact with several distinct pattern recognition receptors, including toll-like receptor 4 (TLR4). These DAMPs, induced by RT, may potentially play an important role in promoting the recruitment, differentiation and effective acquisition, processing and presentation of TAA by stimulating dendritic cell (DC) maturation within TME [21].

Additionally, there are other potential immunological mechanisms through which RT can increase the susceptibility to anti-tumour immune surveillance. These include the multiple death receptors induced by RT expressed on the surface of tumour cells, including FAS (also known as CD95) and tumour necrosis factor-related apoptosis-inducing ligand (TRAIL) receptors 1 and 2 (TRAIL-R1 and TRAIL-R2), which can render the tumour cells more vulnerable to apoptosis [20].

In addition to these immunogenic and phenotypic changes, RT can stimulate immune responses via interactions with the immune system. Whilst the direct contribution of infiltrating versus resident T cells to overall tumour control remains to be clarified, the number and function of TILs have been shown to increase following single, ablative doses of RT. Likewise, both high single-dose and fractionated RT have been shown to increase T cell receptor (TCR) diversity and clonality, predominantly leading to the enrichment of T cell clones already resident within the TME [12,22].

RT can also upregulate MHC class I molecules, leading to enhanced recognition of tumour cells by cytotoxic T cells, and increased NKG2D expression may result in greater NK cell-mediated eradication of tumours [21,23].

Production of type I interferons due to RT-induced accumulation of cytosolic DNA and activation of the STING pathway [24] can also lead to increased expansion of tumour-specific T cells, as a consequence of enhanced cross-presentation of tumour antigens by dendritic cells. RT can trigger the production of pro-inflammatory chemokines including CXCL9, CXCL10 and CXCL16, resulting in the chemotactic recruitment of effector CD8+ T cells into the TME. Macrophages are another immune effector cell population in the TME which may have a potentially important role in dictating the response of the tumour to radiotherapy. Macrophages have been characterised into M1 and M2 phenotypes, although it is understood that there can be “plasticity” between such phenotypes. M1 macrophages mediate vascular normalization, promoting T cell recruitment and subsequent rejection of tumours. In contrast, the M2 phenotype is considered to promote immunosuppression. Interestingly, conventional lower doses of RT (2 Gy) frequently used in routine clinical practice have also been found to transform an immunosuppressive M2 TAM into a tumour-killing iNOS producing M1 TAM [25].

#### 2.2.2. RT-Induced Immunosuppression

**Figure 2:**
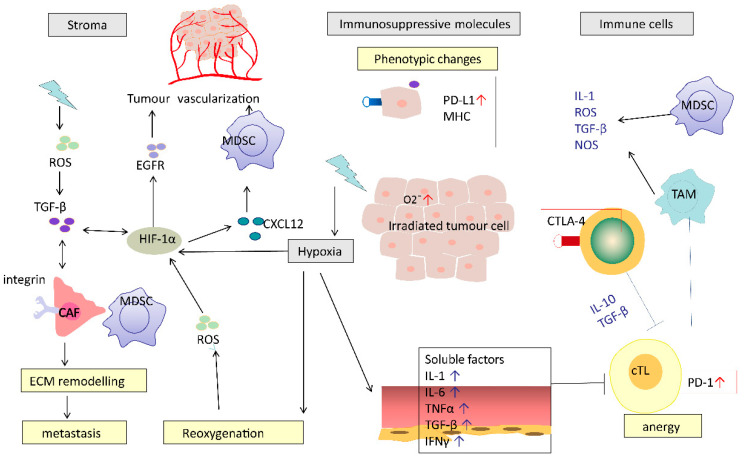
Potential mechanisms of RT-induced immunosuppression. RT causes a series of immunosuppressive processes: modifying tumour stromal environment, production of immunosuppressive molecules, recruiting immunosuppressive cells and impairing effective T cells. RT causes hypoxia, which induces HIF-1α and TGF-β. HIF-1α-activated CAFs mediated by TGF-β or directly by RT promote ECM re-modelling. These effects work in concert to promote cancer metastasis. CAF, cancer-associated fibroblasts; MDSC, myeloid-derived suppressor cell; cTL, cytotoxic T lymphocyte; TAM, tumour-associated macrophages; ECM, extracellular matrix; HIF-1α, hypoxia-inducible factor-1α; TGF-β, transforming growth factor beta.

In contrast to the potential immunostimulatory effects, RT can have several immunosuppressive effects that may negatively impact or suppress the development of anti-cancer immunity. These negative effects of RT can be imposed directly on the TME, such as the RT-induced death of immune cells, or indirectly through modulation of stromal cells and tumour vasculature (Figure 2).

In addition to direct cytotoxic effects on immune cells, RT can lead to the production of pro-inflammatory cytokines and chemokines (such as TNF, interleukin and TGF-β) and the subsequent recruitment of suppressive immune cells such as MDSCs, TAMs and Tregs [26]. TGF-β is a potent immunosuppressive cytokine that inhibits cross-priming of T cells by damaging the antigen-presenting function of dendritic cells and the functional differentiation of T cells into effectors [27]. Infiltration of Tregs can lead to cytotoxic CD8+ T cell inactivation by expression of the checkpoint inhibitor molecule CTLA-4. Interestingly, on some occasions, these immunosuppressive reactions can be overcome by RT-induced immune stimulation, which is characterised by ICD induced antigen exposure, DC maturation, T cell recruitment and activation.

Irradiated normal tissue adjacent to tumour undergoes a process of inflammation, wound healing and fibrosis. This process involves the expansion of cancer-associated fibroblasts (CAFs) and extracellular matrix modelling, which leads to post-radiation tumour hardening and shrinkage, and ultimately facilitates tumour spread or recurrence [26]. CAFs are a heterogeneous cell population that constitute the majority of cells within the stroma in many carcinomas [28]. RT activates CAFs by causing DNA damage and production of ROS [29]. Irradiated CAFs can contribute to cancer progression via the transforming growth factor beta (TGF-β)-CXCL12 dependent pathway. However, whether CAFs play a tumour-killing or tumour-promoting role may vary depending on the type of signal [30]. RT-induced vascular damage is aggravated by tumour hypoxia through CXCL-12 and HIF-1α-mediated MDSC recruitment [31].

In hypoxic environments, RT-induced free radical production is reduced and consequently RT causes less DNA damage in hypoxic areas. A further potentially important mechanism for RT to induce immunosuppression in hypoxic areas may be increases in the production of vascular endothelial growth factor (VEGF), reactive oxygen species (ROS) and hypoxia-inducible factor-1α (HIF-1α) [26]. HIF-1α participates in several specific processes resulting in tumour vascularization and reoxygenation. Furthermore, it was found to be an independent predictor of poor prognosis after RT [26].

#### 2.2.3. RT and Cell Death

Tumour cells usually evolve to escape death by inactivating the cell death pathways commonly used to eliminate damaged and harmful cells [32]. This ability to avoid cell death may lead to resistance during treatment [33]. Therefore, it is necessary to have an overall understanding of the mechanisms of cell death induced by RT in order to develop new anti-tumour treatments. 

For many years, apoptosis has been proposed as the principal cell death pathway induced by radiotherapy. Recently, non-apoptotic cell death morphologies or alternative death mechanisms have been discovered during radiation, including autophagy, necrosis, programmed necroptosis and ferroptosis, all of which will be summarised below. The mode of cell death induced by RT is of particular importance as certain modes of cell death will lead to the release of tumour antigens which are subsequently processed and presented on antigen-presenting cells [21].

Among all these cell death types, one particularly distinct type of “cell death” is radiation necrosis. Radiation necrosis is unprogrammed cell death and, for example, can occur as a rare late side effect following high-dose radiation (typically > 55 Gy) of both intracranial and extracranial tumours, such as nasopharyngeal carcinoma [34]. Unlike all other cell deaths, it has not been often reported to be associated with tumour suppression, but it presents similar imaging findings and symptoms to tumour recurrence, which can pose a challenge to differential diagnosis [35]. The aetiology of radiation necrosis is not yet clear. Possible histopathological changes might be radiation-induced vascular damage and the resultant ischemic necrosis, radiation-induced damage to oligodendrocytes and their precursors or impaired neurogenesis, implicating the vascular cells and/or oligodendrocytes as the targets of radiation injury [34].

Autophagy is a catabolic process whose activation acts as a pro-survival mechanism and can induce autophagic cell death in some circumstances [36]. Targeting autophagy has been proposed as a novel anti-tumour treatment [37]; however, it is still controversial as to whether autophagy stimulation or inhibition is better [38]. Autophagy has also been identified to play a dual role in the response of cancer cells to RT, with preclinical models showing that autophagy inhibition increases radiosensitivity in vitro yet reduces efficacy of radiotherapy in vivo due to deficient immunogenic signalling [39,40]. Inhibiting autophagy with chloroquine or hydroxychloroquine might improve the efficacy of several anticancer therapies in patients, and exploratory clinical studies are ongoing [41]. However, the results of trials using these drugs have been generally disappointing, with limited clinical efficacy [42,43]. In addition, some researchers have proposed that whether autophagy should be enhanced or inhibited is context-dependent [37,44].

Necroptosis is a caspase-independent form of regulated cell death executed by the receptor-interacting protein kinase 1 (RIP1), RIP3, and mixed lineage kinase domain-like protein (MLKL) [45], which can lead to DAMP release [46] and chronic inflammation associated with aging. Much of the tumour radioresistance and recurrence results from tumour cell repopulation after radiotherapy. A preclinical study showed that necroptosis-associated tumour repopulation after radiotherapy depended on activation of the RIP1/RIP3/MLKL/JNK/IL-8 pathway and this novel pathway could be a promising target for blocking tumour repopulation to enhance the efficacy of colorectal cancer radiotherapy [47]. However, there is still a long way to go for necroptosis-based cancer therapy to be developed as many questions are still unclear—for instance, the intrinsic or acquired defects of necroptotic machinery observed in many cancer cells [45].

Ferroptosis is a form of regulated cell death characterised by lipid peroxidation and was recently identified to be potentially tumour-suppressive. Ferroptotic cancer cells have modulatory effects on tumour immunity at many steps [48]; however, various putative signals released by ferroptotic cancer cells may lead to the stimulation or suppression of different immune cells. Triggering ferroptosis may be a novel approach in cancer therapy; several strategies to target ferroptosis have been investigated [48]. Although the link between RT and ferroptosis has not been experimentally addressed or widely proven, recent evidence suggests that radiotherapy induces ferroptosis in cancer patients [49], and increased ferroptosis was demonstrated to be associated with improved response to radiotherapy and better survival in oesophageal tumour samples [50].

## 3. Biomarkers

To date, there have been limited clinical studies addressing the RT-induced immunological changes in humans. Further preclinical and clinical investigations are needed to identify potential biomarkers of RT-induced immunological changes to guide decision-making in clinical practice, particularly when considering RT–IO agent combination therapies.

**Figure 3:**
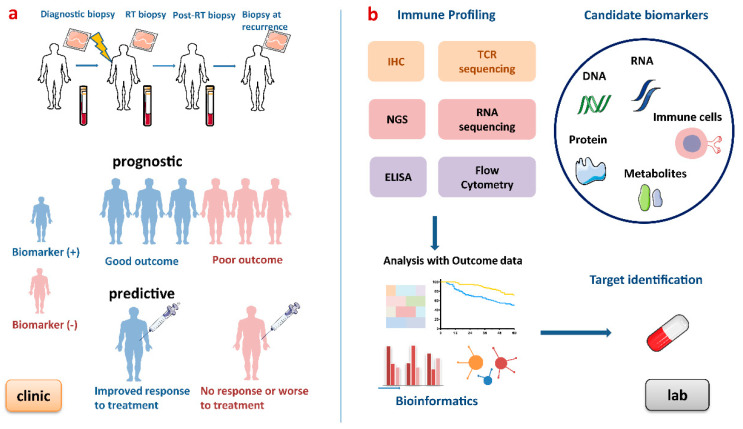
Process of developing biomarkers for patients undergoing radiotherapy. (**a**) Blood and tumour biopsies are taken at baseline, during RT, after RT and at recurrence to obtain dynamic immune profiling. This figure shows an ideal example of prognostic and predictive biomarkers. Prognostic biomarkers provide information on disease progression and outcomes, and predictive biomarkers indicate the response to a medical intervention. (**b**) In the lab, these biomarkers can be discovered by high-throughput gene technologies, immune-based technologies and analysed by bioinformatic approaches. RT, radiotherapy; IHC, immunohistochemistry; NGS, next-generation sequencing; TCR, T cell receptor; ELISA, enzyme-linked immunosorbent assay.

There is growing demand to develop predictive biomarkers which guide the development and delivery of tailored therapies, i.e., “right drug to the right patients”, by predicting benefit to a medical intervention and prognostic biomarkers which are used to identify likelihood of a clinical event, disease recurrence or progression. Several diagnostic tumour markers are now included in routine clinical practice, such as PSA, CA125, β-HCG and AFP [51], but few biomarkers are currently validated for clinical practice in RT and none to guide RT in combination with IO agents. In addition to static biomarkers, dynamic biomarkers are proposed to investigate how treatments may influence key transitions and potentially identify responders and non-responders in a more timely fashion, sparing patients’ toxicities from ineffective treatments and potentially guiding therapy decisions to more effective approaches. Such dynamic tumour biomarkers requiring serial sampling would facilitate real-time decision-making that has not been previously possible with pre-treatment biomarkers [52]. The process of developing biomarkers for patients undergoing RT is outlined below (Figure 3).

### 3.1. Radiotherapy-Related Biomarkers

It is important to note that the TME is dependent on many factors, such as patient age [53], treatment (chemotherapy, radiotherapy and immunotherapy), tumour type (e.g., solid or haematological) and whether the tumour is virally or hormonally driven. For virally driven tumours such as HNSCC, a recent study demonstrated that HPV-positivity correlated with increased immune cytolytic activity and a T-cell-inflamed gene expression profile, suggesting that HPV status can be used to predict the effectiveness of PD-1 inhibitors in HNSCC, independently of PD-L1 expression and TMB, and probably results from an inflamed TME induced by HPV infection and anti-tumour activity of HPV antigen-specific T cells [54]. For hormone dependent tumours such as breast cancer or prostate cancer, the TME may be affected by more complex regulation, such as oestrogen or testosterone signalling pathways [55,56]. Furthermore, it is difficult to delineate whether changes to the TME as a result of RT are due to a specific effect of RT or the change in tumour size as a result of RT, and serial biopsies allowing a thorough investigation into the dynamics and kinetics of changes are only possible in preclinical models. Here, we focus on the impact of RT on the TME, and some of the potential immune-related biomarkers of response to RT which have been identified to date are summarized below (Table 1). Whilst numerous biomarkers during RT are under investigation, there are few available to guide clinical decision-making in predicting responses or in patient selection for radiotherapy-based treatments. Furthermore, biomarker identification is in its infancy and it will be important to consider that any identified biomarker will need to be validated across factors such as different tumour types, age of patients and hormone or viral dependency of the tumour.

#### 3.1.1. PD-1/PD-L1 Expression

Engagement of PD-L1 with its receptor PD-1 results in the inactivation of T cells and NK cells, ultimately contributing to immune evasion [69]. Since Toplian and his colleagues first revealed that tumour PD-L1 expression reflects an immune-active microenvironment [70], several clinical trials have demonstrated that clinical responses to PD-1 blockade are correlated with PD-L1 expression in many cancer types [71,72,73,74].

The role of PD-L1 as a potential biomarker in patients treated with RT has emerged recently [64,75]. The PD-L1 expression after 12 Gy carbon-ion irradiation correlated with better progression-free survival in human uterine cervical adeno/adenosquamous carcinoma [64], while soluble PD-L1 was found to be correlated with worse survival in hepatocellular carcinoma [65]. Interestingly, both studies demonstrated that RT results in increased PD-L1 expression in vivo through interferon gamma production by T cells and that this appears to be an adaptive resistance pathway [10] that can be overcome by PD-L1-blockade [76]. Haematological malignancies such as Hodgkin lymphoma also show upregulation of PD-L1/PD-1 after chemotherapy/radiotherapy [77] and are known to be highly responsive to PD-1 blockade [78], and RT in combination with PD-1 blockade can induce abscopal responses [79]. The Pacific study showed that the addition of durvalumab (anti PD-L1) after consolidated chemoradiotherapy (CRT) enhanced both progression free survival (PFS) and overall survival (OS) of phase III unresectable non-small-cell lung carcinoma (NSCLC) patients; subgroup analysis showed that survival benefit was achieved in both PD-L1 positive and negative patients [76]. Long-term follow-up showed that the survival benefit of durvalumab was observed in subgroups with different PD-L1 levels, except patients with PD-L1 < 1% [80]. The findings from this study were disappointing regarding the potential utility of PD-L1 as a predictive biomarker, but since baseline PD-L1 level before CRT was not mandated and only measured where samples were available, it is currently unknown whether CRT changed the PD-L1 expression level and how it affected the results. Furthermore, durvalumab was given 2–42 days after CRT, which is a quite broad timespan; the optimal time for drug administration should be explored as well as how PD-L1 was affected by CRT dynamically.

In short, the level of PD-L1 within tumours has been suggested to be a promising prognostic biomarker in patients undergoing RT, but the role of PD-L1 as a predictive biomarker still needs further investigation. Ultimately, being able to clearly measure the number of cells expressing PD-L1 (reported as a percentage of stained cells) is critical. This issue is confounded by multiple unresolved problems: variable detection antibodies, differing IHC cut-off values, tissue preparation, processing variabilities, primary versus metastatic biopsies, oncogenic versus induced PD-L1 expression and staining of tumour versus immune cells [81,82]. The cut-off value constituting a positive or negative test result varies by both tumour type and by the antibody clone used to test the sample [83]. Moreover, there are several commercially available IHC assays to measure PD-L1 [83]. The measurement of PD-L1 requires standardization because the PD-L1 can be expressed on both lymphocytes and cancer cells, on both the cell membrane or in the cytoplasm. Recently, an IHC approach was approved by the US FDA as a companion diagnostic to make treatment recommendations for NSCLC patients using the anti-PD-1 agent pembrolizumab [83]. Three additional IHC assays have been approved as complementary diagnostics [84].

#### 3.1.2. Immune Infiltrates

Immune infiltrates were proposed to be reflective of an adaptive anti-tumour response in the TME and may indicate a favourable response to RT. Data on the role of TILs in the context of RT are limited, with mixed findings. A retrospective single centre study examined pre-treatment specimens from 101 neck squamous cell carcinoma (HNSCC) patients before definitive CRT and reported that TILs are associated with favourable survival and are an independent prognostic factor to response to CRT [61]. However, the amount of CD8+ TIL infiltration alone is not always correlated with better clinical outcome. In patients with extrahepatic cholangiocarcinoma treated with adjuvant CRT, high expression of PD-1 on CD8+ TILs was strongly associated with inferior OS and the density of CD8+ TILs alone was not an independent prognostic factor for OS [63].

The presence of TILs can be measured by IHC, flow cytometry and droplet digital PCR technology. Moreover, there is an emerging novel technique of “immuno–positron emission tomography”, which enables identification of CD8+ cells non-invasively and has been used in murine models of melanoma and breast cancer [85]. Whilst flow cytometry may enable quantification of a greater number of immune cells, issues around the requirement for fresh tumours mean that IHC of formalin-fixed tissue biopsies is currently the preferred method of choice for researchers.

#### 3.1.3. Immunoscore

Given that the effect of TILs might be tumour-type-dependent, it is rational to develop combination analysis of different cell populations in immune infiltrates when exploring biomarkers for different cancers. For example, in a retrospective study of 166 CRC cancers with or without preoperative chemoradiation (pCRT), classification into one of five immunoscore groups significantly correlated with differences in disease free survival (DFS) and OS (all *p* < 0.005), with high infiltration of CD3+ and CD8+ lymphocytes in tumour biopsies associated with downstaging of the tumour after pCRT [62]. Currently, immune changes in the TME after CRT, including the value of immunoscore in response to chemoradiation, are mostly found in CRC patients [86]. This is possibly because the immunoscore was first validated and is most studied in CRC, perhaps due to the relatively easy opportunity to obtain biopsies to investigate the effects on the TME after RT and pCRT.

Another study took pre-treatment and post-treatment biopsies from 136 rectal cancer patients who underwent neoadjuvant RT, CT or CRT before radical resection and demonstrated that high levels of CD3+ and CD8+ TILs at baseline were correlated with better pathological responses (TRG ≥ 3) to neoadjuvant CRT and a favourable DFS and OS; in addition, CRT can enhance local immune response by increasing TIL infiltration [87]. One of the difficulties in the interpretation of this study is the diversity of treatment regimens used and the lack of matched immune blood markers to provide correlations with changes in TME. However, despite these caveats, changes in TILs after treatment were not associated with prognosis. Furthermore, Hagland et al. found a correlation between circulating T cells in pre-operative blood with intratumoural density and location of CD3+ and CD8+ T cells in colorectal cancer, suggestive of the potential for a liquid biopsy immunoscore [88]. However, this remains to be investigated for RT responses.

Brain metastases have also proven to be another good opportunity to study the effect of RT after whole brain radiotherapy (WBRT). Berghoff and colleagues investigated the influence of TIL and immunoscore on brain metastases [89]. Interestingly, despite the common preconception that the brain is an immune-privileged site, TIL infiltrates were detected in 115/116 (99.1%) brain metastasis biopsies. The density of CD3+, CD8+ and CD45RO+ TILs showed a significant correlation with favourable median OS. The immunoscore showed significant correlation with survival (27 vs. 10 mo; *p* < 0.001) and was established by multivariable analysis to be an independent prognostic factor (HR 0.612, *p* < 0.001). Interestingly, both the immunoscore and post-surgical WBRT showed an independent additive effect on OS. Considering that WBRT was delivered after surgery, the biopsy could not reflect the impact of RT on the TME; however, the immune infiltrates can serve as baseline data and may aid in patient selection for RT.

#### 3.1.4. Gene Expression Profiling

Genetic mutation is one of the most significant hallmarks of cancer [2] and exists in metastatic settings across many cancer types, particularly frequently in cancers such as bladder cancer, melanoma, NSCLC, CRC and HNSCC [90]. Tumour mutational burden (TMB), which refers to the number of DNA mutations per megabase in a cancer cell, has previously been established as a prognostic biomarker and predictive factor of response to ICI across multiple tumour types [91,92]. The predictive value of gene expression profiling to evaluate the efficacy of RT currently remains unclear. A multicentre retrospective study from Germany developed a 327-gene panel to define TMB and then performed TMB analysis in 101 archival tumour samples from HNSCC patients treated with definitive CRT, aiming to assess the impact of TMB on prognosis. High TMB was demonstrated to be correlated with poor survival, which suggests that it may predict patients who might potentially benefit from CRT-ICI combinations [67]. Further research is needed to determine the value of TMB in response to RT alone.

Another retrospective study performed transcriptome-wide gene expression profiling of primary tumours from 136 muscle-invasive bladder cancer (MIBC) patients treated with bladder-sparing trimodality therapy (TMT) and compared to another cohort of 223 MIBC patients treated with neoadjuvant chemotherapy (NAC) and radical cystectomy (RC) in patients who had not received CRT. Results showed that signatures of T cell activation and IFN-γ signalling were associated with improved disease-specific survival (DSS) in the TMT cohort, but not in the NAC and RC cohort, whereas higher stromal infiltration was associated with shorter DSS after NAC and RC [68]. This suggests that gene expression profiling may be useful in developing predictive biomarkers to CRT but, given the limitations of this retrospective study, additional validation in larger prospective studies is required.

Currently, there is no uniform definition of a high TMB [93]. Standardization of the process of measuring TMB is required, including the method used, the ideal timing of TMB analysis (e.g., diagnostic biopsy or pre-treatment) and the ideal specimen (e.g., primary tumour versus metastasis). DNA next-generation sequencing approaches determining TMB include whole-exome sequencing (WES) and whole-genome sequencing (WGS). WES is considered to be the gold standard test as it generates a large amount of data and gives an overview of the gene mutation landscape; however, the fact that it is costly and requires more DNA may hinder its wider application. Targeted gene panels are unable to cover all the tumour mutations but offer reduced cost and requires less DNA, which enables easier integration into hospital labs.

#### 3.1.5. Neoantigens

Neoantigens, as downstream products of TMB, are small peptide epitopes arising from tumour-specific mutations which are processed and presented on MHC molecules [94,95]. Unlike tumour-associated antigens (TAA) which are commonly shared by patients with the same tumour type, neoantigens are tumour-specific and are generally patient-specific. Recognition of neoantigens may be an important driver of the anti-immune responses to T cell targeting therapy including ICI [95,96,97,98]. One study demonstrated that clinical responses to ICIs mostly occur in patients with pre-existing neoantigen-specific T cells in tumours [99].

There is great interest in developing neoantigens as vaccines to induce immune responses [100]. Although it has been reported that neoantigens were generated in treatment with ICI alone [98] or combined with RT and may be a biomarker of response [66,79], there are some barriers even at the experimental stage. For example, approximately only 1% of all tumour mutations generate a neoantigen with sufficient affinity for MHC to prime T cell responses; therefore, determining how to define a high-quality neoantigen which can trigger robust immune responses still presents a significant challenge. With the development of bioinformatics tools, several findings have emerged. Łuksza and colleagues proposed a neoantigen fitness model based on two main factors: the likelihood of neoantigen presentation on MHC and subsequent recognition by T cells [101]. Another study found that T cells targeting clonal mutations expressed by all tumour cells (trunk mutations) can achieve better anti-tumour immune responses than T cells targeting mutations expressed only in a proportion of tumour cells (subclonal branch mutations) in patients receiving ICI [98]. More work is needed to determine if the number of neoantigens or the frequency of neoantigen-specific T cells may be biomarkers of response to RT with or without ICI.

### 3.2. Imaging Biomarkers

In addition to the above blood-based biomarkers or tissue-based biomarkers, in recent years, non-invasive and clinically useful image-based techniques such as SPE-CT, PET-CT and MRI have been developed to evaluate the immune response to radiotherapy and/or immunotherapy [102]. These advanced imaging techniques may address the dilemma of patients unsuitable for biopisies or with multiple disease sites. A retrospective, European, multicentre cohort study examined tissue samples collected at diagnosis from 208 classical Hodgkin’s lymphoma patients treated with two ABVD (doxorubicin, bleomycin, vinblastine and dacarbazine) courses with fluorodeoxyglucose (FDG)-PET and analysed baseline staging and interim restaging. They found that early-interim FDG-PET scan after two ABVD chemotherapy courses (PET-2) was the only factor able to predict both progression-free survival and overall survival. In PET-2 negative patients, expression of CD68 (≥25%) and PD1 (diffuse or rosetting pattern) in microenvironmental cells, and STAT1 negativity in Hodgkin Reed Sternberg cells, identified a subset of PET-2 negative patients with a 3-year progression-free survival significantly lower than that of the remaining PET-2 negative population [103]. This revealed the combined role of biomarkers and interim PET scan in prediction of treatment outcome and provided a new insight into developing immune cancer biomarkers.

Despite its role in staging being increasingly recognized in lymphoma and non-small cell lung cancer [104], ^18^-FDG–PET has several major drawbacks for use in immune imaging. This is a particular problem when it comes to the phenomenon of pseudoprogression, where the increased immune activity in response to successful therapy is difficult to distinguish from increased tumour metabolism in response to therapeutic failure [105]. Additionally, other limitations of PET imaging are that it is costly and exposes patients to radiation, and these have hindered its wider application in the clinic.

### 3.3. Biomarkers for RT and ICI Combination Therapies

Since the emergence of ICIs as delivering durable anti-tumour responses in a range of cancer types, albeit in the minority of patients treated, the number of clinical trials evaluating immunotherapy alone or combined with conventional oncology treatments such as RT or chemotherapy has greatly increased, with thousands of clinical trials in progress or being developed [106]. Whilst a detailed list of the clinical trials combining RT with immuno-oncology agents is beyond the scope of this review, the synergistic anti-tumour effects of RT and ICI has been investigated in many clinical studies and there are significant numbers of RT and ICI combination trials registered on clinicaltrials.gov [107].

One prospective trial investigated the efficacy of RT plus the anti-CTLA-4 ICI Ipilimumab in relapsed non-small-cell lung cancer. Twenty one of thirty nine enrolled patients completed the treatment protocol, with an overall objective response rate of 18%. Of the 20 evaluable patients, an increased serum IFN-β after RT and early dynamic changes in T cell clones appeared to be the strongest predictive biomarkers of response. Interestingly, T cells targeting a neoantigen controlled by a gene upregulated by RT were identified in one responding patient whose samples were analysed in more detail [66], but this “in-depth analysis” was only performed in one responding patient. Similarly, another study analysed three patients with refractory Hodgkin’s lymphoma treated with a combination of radiation and nivolumab; results showed that all three patients achieved durable complete local and abscopal responses. Further analysis found high PD-L1 expression in all three patient tumours, a mutation in the STAT6 gene, amplification of a “neoantigen” Erb-B2 receptor tyrosine kinase 2 (ERBB2) along with DNA damage response in patient 2 and an intermediate tumour mutation burden (TMB) with 8.15 mutations per megabase in patient 3. This suggested that the expression of PD-L1, DNA damage response and TMB might be potential biomarkers of response [79]. Due to the limitation of a small sample size and the retrospective nature of the study, these findings require validation in larger patient groups.

## 4. Conclusions and Future Directions

Tumours have been broadly divided into immunologically “hot”, characterised by high numbers of tumour-infiltrating T cells, and “cold”, characterised by low numbers of T cells [108]. What is currently less well characterised is how RT interacts with such “hot” or “cold” tumours to induce immune changes in human tumours. RT may well be immunostimulatory and potentially further enhance “hot” tumours and increase the number and diversity of T cell clones or potentially convert a “cold’ tumour to a “hot” tumour with an increase in T cell infiltration. In contrast, RT could be immunosuppressive and convert a “hot” tumour to a “cold” tumour or make the tumour microenvironment of an already “cold” tumour even more immunosuppressive by inducing the cytokines that lead to infiltration of immunosuppressive immune effector cells. An enhanced understanding of how RT affects the TME in “real time” as the patient is receiving RT will be extremely informative but requires a huge effort for serial tumour biopsies with the development of dynamic tumour biomarkers. Only when such data are available can we realistically begin to potentially implement different strategies to potentially enhance or modulate the RT-induced immune response and move towards a more personalized treatment approach for patients.

Currently, preclinical data are emerging which suggest that such a strategy of selecting an IO agent to combine with RT to improve outcomes may be possible at least for the “hot” tumours. In some murine tumours, anti-PD1 (ICI) combined with RT may lead to the generation of systemic immunity and long-term tumour control [10,22]. In contrast, for “cold” tumours, which are normally poorly infiltrated by T cells and contain increased numbers of immunosuppressive myeloid cells, anti-PD1 and RT may be less effective. Therefore, combining RT with IO or immune-stimulatory agents which act to reprogramme myeloid cells and drive increased T cell infiltration into tumours (e.g., TLR agonists, anti-CD40 mAb, anti-OX40 mAb) may be required to overcome this immunosuppressive TME and improve tumour control and survival [109,110].

However, to date, there are still many unanswered questions and future work in both the preclinical and clinical settings is required to establish the optimal RT regimen and any potential biomarkers which may inform IO agent choice when trying to further enhance the synergy of RT in combination with different immunotherapy modalities. Firstly, current data on immune infiltrates and tumour cell PD-L1 expression come from pre-treatment biopsies or surgery specimens, which mainly reflect the immune status at baseline, and there is limited information on changes in TME during or post-treatment. Additionally, few data exist on systemic immune biomarkers post-RT [57,58,59,111]. Therefore, there is an urgent unmet clinical need to collect tumour samples and matching bloods pre- and post-RT from patients undergoing routine RT to further understand the potentially diverse impact of RT between patients and tumour types.

Whilst changes in the TME are likely to be most reflective of immune changes induced by RT, investigations in peripheral blood may provide additional information to enable biomarker detection and surveillance during and after RT with less invasive sampling.

In addition, to identify whether a human tumour is immunologically “hot” or “cold”, there are now many technologies available to analyse immunological markers in tumour and blood. IHC is often used to identify tumour immune contexture by analysing FFPE samples and platforms now exist to multiplex up to 40 different cell markers. RNA gene expression arrays can be used to profile changes in immune genes in greater depth and possible immune gene signatures may arise as potential biomarkers [112,113]. Systemic immune changes can be monitored by profiling of PBMC populations through flow cytometry or mass spectrometry, TCR sequencing to investigate T cell clonality and analysis of serum cytokines and chemokines though multiplex bead-based assays or ELISAs. A thorough investigation of RT-induced changes in the immune TME and in the systemic circulation is the first step to identifying potential biomarkers of immune responses post-RT and determining if these biomarkers are predictive, prognostic or dynamic.

There is still much work to be done to enhance our understanding in the development of biomarkers for RT and RT + immunotherapy combinations in the clinic. Although there are currently thousands of clinical trials evaluating IO agents alone or combined with conventional oncology treatments such as RT or chemotherapy [58], only the minority have translational immunological research that may provide mechanistic insights and help the development of immunological biomarkers. For RT and RT in combination with IO agents, this field is still in its infancy and requires the coordinated efforts of the pioneers to incorporate biomarker-driven research into their design. Only with this focus on translational research will progress be made and provide us with the possibility that tumours may be stratified for treatment according to immune biomarkers identified in the TME and/or the peripheral blood that will predict immune responses post-RT and may inform RT/IO combinations.

## Figures and Tables

**Table 1:** Clinical studies on immune-related biomarkers in radiotherapy-based treatment.

Category	Sample Origin	Biomarker	Cancer Type	Treatment	Clinical Response	Reference
Circulating cells	Peripheral blood mononuclear cell	Lymphopenia	Cervical cancer	Definitive CRT	↓ OS, DFS	[57]
	Whole blood	↑ Myeloid-derived suppressor cell	HCC	RT	↓ OS	[58]
	Whole blood	↑ MDSCs + Tregs	Rectal cancer	Short-term pCRT + surgery	Early marker of response	[59]
Circulating cytokine	Plasma	VEGF, PIGF and IL-6	CRC	Neoadjuvant bevacizumab + CRT + surgery	Predictive of response	[60]
Immune Infiltrates	FFPE tissue	↑ CD3+, CD8+ TILs	HNSCC	Definitive CRT	↑ OS, PFS Predictive of CRT	[61]
	FFPE tissue, surgical specimens	↑ CD3+, CD8+ TILs	CRC	pCRT	Predictive of pathological downstaging	[62]
	FFPE tissue	↑ Ratio of PD-1+/CD8+ TILs	Extrahepatic cholangiocarcinoma	Adjuvant CRT	↓ OS	[63]
	FFPE tissue, surgical specimens	Immunoscore	CRC	pCRT	Predictive of downstaging after pCRT	[62]
	Surgical specimens	Immunoscore	Brain metastasis	Surgery + whole brain RT	Prognostic to OS	[62]
T-cell-related	FFPE tissue	PD-L1 (+) post RT	Cervical carcinoma	Carbon-ion	↑ PFS	[64]
	Plasma	↑ PD-L1	HCC	RT	↓ OS	[65]
	Plasma	T cell receptor repertoire and ↑ serum IFNβ	Lung cancer	RT + ipilimumab	Predictive of response to combination therapy	[66]
Gene expression profiling	FFPE tissue	High tumour mutational burden	HNSCC	Definitive CRT	↓ OS	[67]
	FFPE tissue	↑ IFN-γ signature	Bladder cancer	pCRT + surgery + CRT	↑ Disease-specific survival	[68]

CRT, chemoradiotherapy; RT, radiotherapy; HCC, hepatocellular carcinoma; pCRT, preoperative chemoradiotherapy; CRC, colorectal cancer; FFPE, formalin-fixed paraffin-embedded; HNSCC, head and neck squamous cell carcinoma; PD-L1, programmed death-ligand 1; OS, overall survival; PFS, progression-free survival; DFS, disease-free survival; ↑ increased; ↓ decreased.
